# Confronting Earth System Model trends with observations

**DOI:** 10.1126/sciadv.adt8035

**Published:** 2025-03-12

**Authors:** Isla R. Simpson, Tiffany A. Shaw, Paulo Ceppi, Amy C. Clement, Erich Fischer, Kevin M. Grise, Angeline G. Pendergrass, James A. Screen, Robert C. J. Wills, Tim Woollings, Russell Blackport, Joonsuk M. Kang, Stephen Po-Chedley

**Affiliations:** ^1^ National Science Foundation National Center for Atmospheric Research, Boulder, CO, USA.; ^2^ The University of Chicago, Chicago, IL, USA.; ^3^ Department of Physics, Imperial College London, London, UK.; ^4^ University of Miami, Miami, FL, USA.; ^5^ ETH Zurich, Zurich, Switzerland.; ^6^ University of Virginia, Charlottesville, VA, USA.; ^7^ Cornell University, Ithaca, NY, USA.; ^8^ University of Exeter, Exeter, UK.; ^9^ University of Oxford, Oxford, UK.; ^10^ Canadian Center for Climate Modelling and Analysis, Environment and Climate Change Canada, Victoria, BC, Canada.; ^11^ Lawrence Livermore National Laboratory, Livermore, CA, USA.

## Abstract

Anthropogenically forced climate change signals are emerging from the noise of internal variability in observations, and the impacts on society are growing. For decades, Climate or Earth System Models have been predicting how these climate change signals will unfold. While challenges remain, given the growing forced trends and the lengthening observational record, the climate science community is now in a position to confront the signals, as represented by historical trends, in models with observations. This review covers the state of the science on the ability of models to represent historical trends in the climate system. It also outlines robust procedures that should be used when comparing modeled and observed trends and how to move beyond quantification into understanding. Finally, this review discusses cutting-edge methods for identifying sources of discrepancies and the importance of future confrontations.

## Teaser

The science of confronting Earth System Model trends with observations is reviewed, and future directions are discussed.

## INTRODUCTION

The climate system is rapidly evolving under anthropogenic forcings and will continue to do so over the coming decades. For over 40 years and through several rounds of Intergovernmental Panel on Climate Change (IPCC) reports, the climate science community has made projections of climate change under specified future emissions scenarios using Climate models or Earth System Models (ESMs). Considerable effort is made to ensure that these models represent the climate system with fidelity, but large uncertainties in future projections still exist, especially at the regional scale (*1*).

Multiple factors can contribute to long-term trends in the climate system. In recent decades, the relative role of anthropogenic forcings, such as greenhouse gases and aerosols, has increased. The climate system responds to these forcings directly but also modifies and modulates the impact of these forcings through various feedbacks, while natural forcings as well as internal variability of the climate system can also contribute to long-term trends. The ocean, in particular, exhibits notable variability on interannual and multidecadal timescales, including in the tropical Pacific and North Atlantic, respectively. Via atmospheric and oceanic teleconnections, these regions can have remote impacts elsewhere. But the random sampling of higher-frequency internal atmospheric variability can also contribute to historical trends in observations, or in individual model simulations. The trends at any given location of any particular feature of the climate system can, therefore, be affected by both the direct response to external forcings, local and nonlocal feedbacks in response to those forcings, and the effects of internal variability of various timescales and origins, i.e., many processes that must be adequately represented in ESMs.

Validating ESMs’ ability to accurately simulate the climate response to external forcings (i.e., the signal) has historically been challenging due to short and/or uncertain observational records, large uncertainties due to internal variability, and a relatively small forced signal. These challenges have set climate prediction apart from weather prediction, although the two rely largely on similar numerical models and physics. Numerical weather prediction (NWP) can be validated on rapid cycles, allowing for a prompt identification of model shortcomings and targeted model improvements—resulting in clear improvements in NWP skill over recent decades (*2*). By contrast, it takes years or decades to accumulate observations of sufficient length to evaluate model projections of future trends. High-quality observations are not always available to validate model simulations of the historical era, or if they are available, the length of record can be rather short in the face of the internal variability in the system, which can manifest as multidecadal trends. Consequently, compared to NWP, with the exception of some work on seasonal-to-decadal predictions, climate prediction has seen a greater reliance on the understanding, validation, and improvement of physical climate processes [e.g., (*3*, *4*)], in the absence of opportunity to truly validate model skill by comparing predictions to what actually happened.

Through the coordinated efforts of the Coupled Model Intercomparison Project (CMIP), which was established in 1995, global numerical models of the climate system (referred to as ESMs or models hereafter) are routinely used to simulate the evolution of the climate system starting from “pre-industrial” times (taken to be 1850) and then evolving until near present day under prescribed time-evolving observation-based external forcings, including greenhouse gases, anthropogenic and naturally produced aerosols, volcanic, solar, and ozone-depleting substances and/or ozone, as well as land-use and land-cover change (*5*–*7*). These ensembles include uncertainty due to internal variability, model structural, and scenario uncertainty (*1*). Individual groups have also produced large ensembles of simulations following similar protocols to CMIP, allowing the forced response and the internal variability to be assessed in individual models (*8*–*12*). The result is a wealth of model predictions that can be used to probe whether the historical trends simulated by ESMs are in agreement with observed trends, as well as to understand the mechanisms involved and the relative roles of external forcing and internal variability.

We are now in 2025, the planet has warmed on average by around 1.2°C since 1880, and 2024 was the warmest year on record to date at 1.47°C above the 1850–1900 baseline (*13*), surpassing the previous record that was set in 2023 (*14*). For many features of the climate system, we are now living in a world where the climate change signal is clearly apparent. Furthermore, satellite-based observational records are now over 45 years long and in situ measurements extend even further back in time. Observational capabilities have advanced, and models too have increased in the range of complex processes that they represent. Model simulation strategy has also evolved: Large ensembles are now commonplace, which enables accurate quantification of the modeled forced response and the potential impact of internal variability on historical trends.

While challenges still remain in isolating forced signals from internal variability, the confluence of the growing climate change signal, the lengthening observational record, and technological advances in both observations and modeling means that the climate science community is now uniquely positioned to confront historical trends in models with those in the observational record and address: What are we getting right? What are we getting wrong? Why? What is still challenging to assess?

The focus of this review is the comparison of observed historical trends to those in global ESM simulations that are forced by time-varying observation-based external forcings, whereby “historical” trends refer to changes over any segment of time within the instrumental (1850 to present day) period, as used in the cited studies. The review will discuss where the field of climate science stands in its comparison of observation-based historical trends to model simulations by summarizing some of the major discrepancies and successes that have been found so far. The distinction between a success and a discrepancy can depend on the definition of success. For example, as will be outlined below, there are many features where models have been highly successful at capturing the pattern of changes that have occurred over the observational record, but in some cases, they are misrepresenting the magnitude of this change. This can be considered to be a success in that models have accurately predicted the direction of climate system change, which allows the climate science community to develop theories for the origin of that change and identify spatial fingerprints to detect and attribute trends in the observational record to anthropogenic forcing (*15*). However, when it comes to quantitatively using our models to predict the future, this mismatch in magnitude represents a discrepancy that we must understand and resolve.

The identification of a success or a discrepancy also often involves some degree of subjectivity and often requires expert judgment on the level of discrepancy based on where the observations lie within the spread of model simulations, and their confidence in the fidelity of the observations themselves. We, therefore, also provide recommendations on best practices and procedures when comparing modeled trends with observations and some priorities in terms of moving beyond quantifying discrepancies (or otherwise) to actually understanding how the processes are being represented in models. This will involve discussion of new tools and methodologies that may be developed and applied in the future. We will close by proposing some priorities for the future to ensure that climate models not only represent historical trends with fidelity but also provide accurate projections for the future of the climate system.

## ACCUMULATING SUCCESSES AND DISCREPANCIES

The field of climate science has now accumulated an array of successes, discrepancies, and uncertainties when it comes to the ability of ESMs to represent historical trends (*16*). We summarize some of these cases in Table 1. The successes (S) are examples where there is a substantial trend in the observed record and both the sign and magnitude of that trend are well represented in most models; the partial successes (PS) are those where there is a substantial trend in the observed record and the sign and structure of that trend are well represented in models but the magnitude is not; the discrepancies (D) are cases where there is a substantial trend in the observations and/or the models but they disagree; and the uncertain trends (U) are cases where there are concerns that either models may have captured historical trends for the wrong reasons, where internal variability makes it challenging to draw firm conclusions, or where there is a substantial concern about the fidelity of the observational record.

**Table 1.** Summary of literature comparing modeled and observed historical trends. The first column lists the field, the second column summarizes the conclusions that have been drawn for this field, the third column lists some relevant references, and the fourth column lists whether the ability of models to represent trends in this field represents a success (S), a partial success (PS), a discrepancy (D), or whether the situation is uncertain (U).

Quantity	Summary	References	
Global mean temperature	Most models accurately represent long-term historical global mean temperature rise over the instrumental record, although they tend to do so with greater warming trends in the tropical Pacific than observed (Fig. 2).	(*19*) and figure 1.9 of (*21*)	S
Global column water vapor	Models accurately represent the historical rise in globally averaged column integrated water vapor.	(*51*, *213*)	S
NH summer jet stream and storm track trends	Models accurately represent the observed weakening of the NH summer jet stream (Fig. 1A). The CMIP6 models also capture the weakening of the NH summer storm tracks but CMIP5 did not.	(*69*, *72*, *214*)	S
Marine heat waves	Models capture the increasing probability of marine heatwaves over the satellite era.	(*44*, *45*)	S
Amplitude of the SST seasonal cycle in the Northern Hemisphere	Models capture the observed increase in the amplitude of the seasonal cycle of SSTs in the Northern Hemisphere.	(*39*)	S
Increasing intensity of extreme precipitation events (global)	Models and observations broadly agree on increasing trends in extreme precipitation intensity when aggregated globally. Uncertainties are larger for regional patterns, and whether the globally aggregated trends in the magnitude of the increase in intensity is a success can depend on the metric used.	(*61*–*64*)	S
Pause of SH circulation trends as ozone recovers	Models capture a poleward shift of the SH mid-latitude jet in response to ozone depletion and also represent a pause in that shift as ozone starts to recover—both of which are also seen in observations.	(*68*)	S
Hadley cell extent	The expansion of the tropics as measured by the Hadley cell edge lies within the modeled distribution of trends, as long as it is calculated using well-constrained surface metrics in modern reanalyses. Previously documented discrepancies were resolved by using newer-generation reanalyses, considering surface metrics of the Hadley cell edge, and accounting for internal variability.	(*58*)	S
Wintertime cold extremes in the NH	Earlier studies argued that an increase in observed cold extremes was different from model behavior. But updated analysis accounting for temporal variations in observational coverage now indicates that models and observations agree in a decline of NH cold extremes during winter (Fig. 3).	(*47*)	S
Arctic warming	The observed warming of the Arctic during the satellite era lies within the modeled distribution of trends.	(*20*)	S
Tropical overturning circulation	Both models and observations exhibit a weakening of the global overturning circulation over the historical record, but the magnitude of this weakening in the tropics is overestimated in models. There are also local discrepancies such as in the tropical Pacific.	(*52*–*54*)	PS
Contrast between tropical dry and wet regions	Precipitation contrasts between dry and wet regions in the tropics have increased. Models represent this, but the magnitude of the observed change is larger than most model simulations.	(*55*)	PS
Increased precipitation variability	Models show an increase in precipitation variability, which has now been observed, although there may be discrepancies in magnitude in some regions.	(*67*)	PS
Tropical tropospheric temperature	Models and observations agree on historical warming of the tropical troposphere, but the warming in most model simulations is too large. Recent studies suggest a likely role for the combined influence of internal variability, discrepancies in the tropical warming pattern, issues with the forcings provided to models, too-large climate sensitivity in some models, and observational biases.	(*31*, *36*, *37*, *215*)	PS
TOA radiative imbalance	Models and observations both exhibit an increasing trend in TOA radiative imbalance, but the magnitude of the trend since 2001 is underestimated in models compared to observations.	(*25*, *26*)	PS
Arctic amplification	Models robustly predict amplified warming of the Arctic compared to elsewhere, which has been observed. Models also seem to capture the magnitude of the warming of the Arctic (see success above), but there are concerns that models may be underestimating the magnitude of this amplified warming relative to warming in the rest of the planet, particularly in recent decades when internal variability is thought to have enhanced Arctic amplification trends in observations.	(*23*, *24*, *176*, *216*, *217*)	PS
Arctic sea ice	Models capture the observed declining trend, but internal variability leads to a large uncertainty and is thought to have contributed to the magnitude of the observed decline. There are indications that models may be capturing sea ice trends for the wrong reasons.	(*22*, *218*–*220*)	PS
Tropical SST pattern	Most model ensemble members fail to capture the observed strengthening of the tropical Pacific SST gradient and instead predict a weakening (Fig. 1B). This is true both in a narrow band at the equator and for the broader tropical pattern.	(*53*, *73*–*75*, *126*)	D
Wintertime North Atlantic jet	Models fail to capture the observed strengthening of the North Atlantic jet and associated impacts on European precipitation since 1951.	(*88*)	D
JJA Greenland blocking	The recent increase in Greenland blocking events seen in observations is not captured in model simulations.	(*89*, *90*)	D
Exacerbated summer warming in western-central Europe	Western-central Europe has seen exacerbated warming and drying compared to the global mean and a substantial rise in heat extremes that is not well captured by models.	(*91*–*94*)	D
Arid region near-surface specific humidity	Models suggest that near-surface specific humidity in arid regions should have risen over the historical record. A rise has not been observed.	(*85*–*87*)	D
Southern Ocean SSTs and sea ice extent	It is rare for model ensemble members to reproduce the observed slight decline in Southern Ocean SSTs and increase in Southern Ocean sea ice extent since 1979, although rapid declines in Southern Ocean sea ice have been observed in recent years.	(*53*, *79*)	D
Winter Eurasian cooling/warming hole	The observed winter cooling or suppressed warming over central Eurasia is within the range of modeled internal variability, but it has also been argued that the forced response in models could be too weak.	(*98*–*102*)	U
Hadley circulation strength	Reanalyses exhibit a strengthening, while climate models exhibit a weakening, but there are indications that the reanalyses are in error.	(*59*, *60*, *221*)	U
SH storm track	Chemke *et al*. (*103*) showed that the SH storm track strengthening in certain reanalyses is greater than in models, but using a wider array of reanalyses and like-for-like comparison, Kang *et al*. (*84*) demonstrated a large observational uncertainty (Fig. 1C) and that the discrepancy may be smaller than originally thought and likely influenced by discrepant tropical Pacific SST trends.	(*84*, *103*, *222*)	U
Zonal mean jet stream	There are indications that models are adequately capturing the poleward shift of the jet streams but that they may not have the correct relationship between upper tropospheric warming and this poleward shift, so this may be for the wrong reasons.	(*104*)	U

When a discrepancy is found between observed and modeled trends, it could arise for multiple reasons [(*17*), see also the “Robust Procedures for Confronting Observed and Modeled Trends” section]:

1) The simulated response to the observed external forcings could be in error.

2) Internal variability in the models may be misrepresented (e.g., if internal variability is too small, the distribution from a model may not encompass the observed trend even if the forced trend is well represented).

3) The observation-based external forcings prescribed within the model may be misrepresented.

4) The observational record may underestimate or lack uncertainty information and may contain unresolved or undiagnosed biases.

5) There could be issues with the methods that are used to perform the comparison against observations.

Even in cases where there is good agreement between models and observations, it is possible that further investigation will indicate that this agreement may not be for the right reasons. There are examples of each of these in the recent literature that we now summarize. We begin with the many successes and partial successes related to large-scale thermodynamic trends, temperature variability and extremes, the hydrological cycle and overturning circulation, and jet streams and storm tracks while also mentioning relevant discrepancies and uncertainties that are directly connected to these features in the first four subsections and then we summarize the major current known discrepancies in the fifth.

### Successes and partial successes in large-scale thermodynamic trends

Many of the large-scale thermodynamic aspects of historical trends that have occurred in response to rising greenhouse gases are successfully or partially successfully represented in models. Current state-of-the-art models and even the coarse-resolution climate models (by today’s standards) of the 1970s and 1980s successfully predicted the observed rise in global mean temperature and its spatial structure, including the amplified warming of the Arctic and the greater warming over land (*18*–*20*) [figure 1.9 of (*21*)]. While the representation of the pattern of enhanced warming of the Arctic is a robust success for the models, related concerns have emerged. For example, Rosenblum and Eisenman (*22*) found that while the CMIP5 models were able to reproduce historical trends in Arctic sea ice, they may not be doing so for the right reasons given that it was only the simulations with larger global warming trends than observed that were able to capture the observed magnitude of sea ice decline. There are also concerns that models may be underestimating the magnitude of the Arctic amplification relative to observations, particularly for the recent decades (*23*, *24*). The success of models in capturing the trends in top-of-atmosphere (TOA) radiative balance that ultimately is the driver of, and a response to, surface temperature changes is also currently only considered to be a partial one. While models display a robust rise in the TOA radiative imbalance, the magnitude of that trend since 2001 seems to be underestimated in models (*25*, *26*).

Early climate model simulations and present-day climate models consistently produce several other temperature signals in response to greenhouse gas increases, including stratospheric cooling and tropospheric warming, with enhanced warming in the tropical upper troposphere relative to the surface and the aforementioned polar amplified warming (*27*, *28*). Early assessments of tropospheric temperature trends derived from satellite and radiosonde observations conflicted with model expectations, because they exhibited limited warming compared to surface temperature datasets (*29*). As tropospheric temperature datasets improved, observations began to support expected changes to the thermal structure of the atmosphere including substantial tropospheric warming, with enhanced warming in the tropical upper troposphere and Arctic lower troposphere, and stratospheric cooling [e.g., (*30*, *31*)]. Despite these successes, the level of model-observational agreement with regard to atmospheric temperature change is sensitive to the time period, metric, dataset, and region considered (*31*). Tropical tropospheric warming has been persistently controversial, because satellite and radiosonde datasets tend to have smaller trends than expected given the rate of tropical surface warming and smaller trends than most model simulations (*32*, *33*). A recent update to one dataset that showed the most tropospheric warming (National Oceanic and Atmospheric Administration Center for Satellite Applications and Research or NOAA STAR) resulted in a large reduction in the estimated warming, further exacerbating model-observational differences, but also illustrating the large structural uncertainty inherent in the construction of these records (*34*, *35*). Model-observational discrepancies in tropical tropospheric warming are likely due to a combination of factors including a reduction of observed warming, compared to the forced signal, due to internal variability, biases in the forcing prescribed to models, and residual observational biases, and because some models have climate sensitivity values that are likely too large (*36*–*38*).

### Successes and partial successes in temperature variability and extremes

Observations have exhibited an intensification of the seasonal cycle of sea surface temperature (SST) in the Northern Hemisphere (NH) mid-latitudes, which has also been captured in models (*39*). In many places, models are also representing the rise in temperature extremes over land reasonably well (*40*–*43*), as well as the rise in marine heatwaves in the ocean (*44*, *45*), although recent work indicates that models may be missing an observed stretching of the tail of summertime cold extremes (*46*). For earlier work on wintertime cold extremes, issues with analysis methods and, in particular, not performing a “like-with-like” analysis in the presence of changing data coverage over the observational record has recently been shown to play an important role in past conclusions (*47*). It had previously been argued that models were poorly representing a rise in observed NH cold extremes (*48*). This observed trend was contrary to expectations given the substantial warming of the Arctic where the air that contributes to cold extremes originates (*49*). Blackport *et al*. (*47*), however, have now found that the previously reported increase in observed cold extremes arose because prior research had not accounted for changes in the observational data coverage. Once this is accounted for, the models and observations are brought into better alignment, also discussed further in the “Robust Procedures for Confronting Observed and Modeled Trends” section.

### Successes and partial successes in hydrological cycle and overturning circulation

Theories predict that, alongside global warming, on average, there should be a rise in global mean atmospheric humidity and both a weakening of the atmospheric overturning circulation and an enhanced hydrological cycle contrast between wet and dry regions: the so-called wet-get-wetter, dry-get-drier paradigm (*50*). Models successfully represent the rise in global mean atmospheric humidity (*51*) but with regional exceptions discussed further below. The success of models in reproducing the weakening of the atmospheric overturning circulation and the enhanced hydrological cycle contrast between wet and dry regions is partial. They have predicted these robust features of climate change and have allowed the climate science community to develop theories around them. The observational record now shows signs of both a weakening of the global and tropical overturning circulation and of an increasing precipitation contrast between dry and wet regions, but there are concerns that the magnitude of the weakening of the tropical overturning in models is too large (*52*) and locally there can be discrepancies such as in the tropical Pacific where models predict a weakening of the Walker circulation and the observations show a strengthening in recent decades (*53*, *54*). Perhaps related to the difference in magnitude of the overall weakening of the tropical overturning circulation, there are also concerns that the magnitude of the increasing contrast between dry and wet regions in models is too small (*55*), although it remains to be seen whether this conclusion is robust to updated precipitation datasets.

Models and observations are now considered to agree on historical trends in the expansion of the tropics as represented by a poleward shifting of the edges of the Hadley circulation. But this has not always been the case. Early studies documented much larger tropical expansion rates than represented in models (*56*, *57*), but with newer reanalysis products and updated satellite and observational datasets, the use of more observationally constrained surface metrics of the edge of the tropics in reanalyses, and a quantification of the uncertainties due to internal variability through large model ensembles, the differences between models and observations in the rates of tropical expansion have now been shown to be minimal (*58*). For trends in Hadley circulation strength, the picture remains uncertain as reanalyses tend to exhibit a Hadley cell strengthening while models exhibit a weakening, but there are indications that the reanalyses may actually be in error (*59*, *60*), emphasizing the challenges with fields like the divergent circulation in the tropics where the observational constraints for reanalyses can be lacking.

Models and observations both exhibit an increase in the intensity of precipitation extremes with reasonable agreement when aggregated across the globe, although the level of success can depend on the metric used, especially in terms of the agreement on magnitude of increase (*61*–*65*). Aside from consideration of changes in extreme events, models have also indicated that the temporal variability of precipitation more generally should also increase with warming (*66*) and this is a feature that is now being seen in observed trends, although with indications that the magnitude of the trend may be smaller than in models (*67*).

### Successes and partial successes in jet streams and storm tracks

**Fig. 1.**
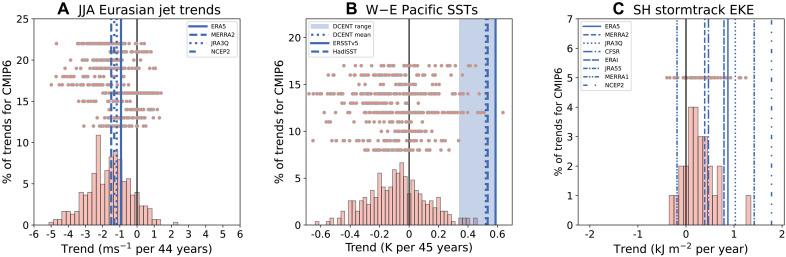
Three examples of trends over the historical record. (**A**) Success. (**B**) Discrepancy. (**C**) Uncertain situation. (A) The 1980–2023 trends in the summertime Eurasian jet stream following the method of (*69*). It shows the trend in June, July, and August (JJA) area averaged 200-hPa zonal wind over 30°E to 120°E, 35°N to 45°N for (salmon pdf) 303 members from 41 CMIP6 models, (salmon dots) from bottom to top: five CMIP5-era large ensembles (CanESM2, CESM1-CAM5, CSIRO-Mk3-6-0, GFDL-CM3, and MPI-ESM) and six CMIP6-era large ensembles (ACCESS-ESM1-5, CanESM5, EC-Earth3, MIROC6, MPI-ESM1-2-LR, and CESM2). Observation-based data are shown in blue. (B) The 1979–2023 trends in annual mean difference between SST averages over 5°S to 5°N in the Western Pacific (110°E to 180°E) and Eastern Pacific (180°E to 280°E) as in (*53*) for (salmon pdf) 271 members from 41 CMIP6 models and (salmon dots) the same large ensembles as in (A). Observation-based data are in blue, and the blue range shows the minimum to maximum range of the 200-member ensemble of the DCENT observational dataset (*76*). (C) The 1979–2018 trends in SH winter (June-July-August) storm track activity as measured by 2.5- to 6-day band-pass filtered eddy kinetic energy vertically integrated and averaged over 40°S to 75°S for one member from 26 CMIP models and the CESM2 large ensemble [data taken from (*84*)].

Ozone depletion has been an important driver of trends in the Southern Hemisphere (SH) spring and summer, and models were successfully able to predict the poleward shift of the SH westerlies that arose as a result of ozone depletion and also the subsequent stalling in SH circulation trends that has now occurred as ozone begins to recover (*68*). Aerosols have also played an important role in NH circulation changes, and a robust weakening of the Eurasian summertime jet stream has been identified, which is successfully captured by ESMs and can be attributed to anthropogenic aerosols (*69*). Regional trends in summertime storm track intensity over the North Pacific can also be attributed to anthropogenic aerosols (*70*). The successful prediction of summertime Eurasian jet trends is depicted in Fig. 1A following the methods of (*69*) but extending the trends out to 2023 and incorporating a larger number of model simulations. The models indicate a forced weakening of the Eurasian jet that aligns well with what has been observed, but the large ensembles also illustrate the large range of potential histories we could have observed as a result of the combined influence of this forced signal and of internal variability. Comparison of the spread from individual large ensembles with that across the CMIP6 models demonstrates the importance of internal variability for the overall spread of the CMIP6 multimodel ensemble.

A weakening of the zonal mean summertime storm tracks has also been observed, and while CMIP5 models were unable to capture the observed trends (*71*), CMIP6 is improved, which has been argued to be related to a difference in the prescribed aerosol forcings (*72*). So this is an example where the models may not have been in error but actually the forcings that were given to the models may have been in error, although the role of the forcings has so far only been rigorously demonstrated within one model and it remains to be seen whether this can explain the difference between CMIP5 and CMIP6 more generally.

### Current discrepancies and uncertainties

Despite these many successes or partial successes, there are many areas where discrepancies between observed and modeled historical trends are now emerging and some have already been discussed in relation to the features described above. The most prominent of these is a discrepancy in the trends in tropical Pacific SSTs. Most ESMs suggest that over the last few decades, the effects of anthropogenic forcing should have resulted in a relative warming of the eastern tropical Pacific and a weakening of the zonal gradient in SST across the Pacific. In contrast, the observational record has exhibited the opposite: a relative cooling of the eastern tropical Pacific and a strengthening of the zonal SST gradient. Despite the importance of internal variability in this region, very few model simulations capture the observed trends (*53*, *73*–*75*). This difference in tropical Pacific SST trends is likely linked to the discrepancy in Walker circulation trends discussed above. This discrepancy in the trend in the difference in warming between the west and east Pacific is reproduced in Fig. 1B, where out of the 495 simulations considered, only 1 exhibits a trend as positive as the mean of the Dynamically Consistent Ensemble of Temperature (DCENT) observation-based ensemble (*76*). Even considering the minimum end of the observational uncertainty range as represented by the DCENT ensemble, only 20 of 495 simulations exceed that minimum trend.

**Fig. 2.**
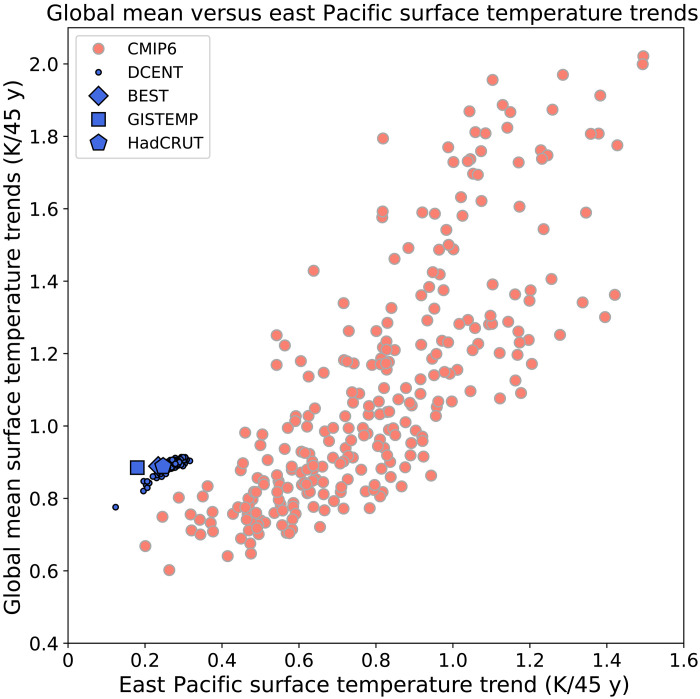
Relating global mean temperature trends to eastern tropical Pacific temperature trends. The 1979–2023 trends in global mean surface temperature versus surface temperature in the East Pacific (180°E to 280°E, 20°S to 20°N), following (*77*). The salmon points show 271 members from 41 CMIP6 models, and the blue points show observation-based datasets. For the CMIP6 models, SST (ts) is used over ocean and surface air temperature (tas) is used over land (see Materials and Methods).

The discrepancy in tropical Pacific SST trends is likely affecting global mean temperature trends (*77*, *78*). The connection between the global mean temperature and tropical Pacific SST trends was first highlighted during the global warming hiatus period from 1993 to 2012. An updated trend analysis following (*77*) but for a longer time period (1979–2023) demonstrates the clear relationship between the rise in eastern tropical Pacific SSTs and the rise in global mean temperature across the model ensemble. Note that the Eastern Pacific region used here is broader than the localized region that has exhibited a cooling; averaged over this broader region, there is a weak overall warming in observations. This analysis suggests that excessive warming of the eastern tropical Pacific in models is likely contributing to excessive global mean warming in the models. The four observational datasets, however, do not lie within the bivariate model distribution, exhibiting a global mean rise in temperature that is larger than would be expected based on the model bivariate distribution and the observed Eastern Tropical Pacific SST trends (Fig. 2).

There is growing evidence of a connection between the discrepancy in eastern tropical Pacific SST trends and a discrepancy in Southern Ocean SST trends, where models also suggest the Southern Ocean should have warmed, in contrast to the slight cooling that has been observed (*53*, *79*). Models also suggest that Antarctic sea ice should have declined, in contrast to the long-term rise that was seen until recent years (*79*) when Antarctic sea ice area suddenly contracted (*80*). Idealized experiments in which the Southern Ocean is cooled also produce cooling in the lower latitude South Pacific (*81*, *82*), and there is evidence that increasing resolution enhances multiyear initialized prediction skill in both the Southern Ocean and the Tropical Pacific, with the Southern Ocean leading the Tropical Pacific (*83*). The atmospheric circulation also couples SST trends in the tropics to the South Pacific (*84*). These findings all point to a link between the trends in the Southern Ocean and those in the Tropical Pacific in both directions. There are, however, also arguments for potential tropical origins of this discrepancy in equatorial Pacific SST trends related to biased mean states in these regions (*74*). This discrepancy in tropical Pacific SST trends likely has global consequences and may be related to some of the aforementioned discrepancies in the magnitude of changes such as that in TOA radiative imbalance or the meridional overturning circulation in the tropics.

Other regional discrepancies have also emerged. While models accurately represent the rise in global mean vertically integrated humidity (*51*), they do not capture the observed declines in continental near-surface relative humidity (*85*, *86*). In arid and semi-arid regions, the discrepancy is particularly salient, with models showing a rise in near-surface specific humidity that is close to Clausius Clapeyron scaling and observations showing no rise at all on average (*87*).

The North Atlantic and western Europe are also subject to a number of trend discrepancies. Models fail to capture the observed strengthening of the wintertime North Atlantic jet and associated impacts on European precipitation since 1951 (*88*). In the summertime, there has been a recent rise in the occurrence of Greenland blocking events, which is not captured in model simulations (*89*, *90*). Western-central Europe has also experienced exacerbated summertime warming and drying compared to the global mean. This has been associated with a rise in heat extremes that exceeds the increase typically found in models and has been argued to be linked to circulation changes (*91*–*93*). In contrast, the midwestern United States has experienced a muted warming trend in annual temperature maxima that is smaller than in most models and has been related to a combination of unforced internal variability (*94*) and cropland intensification and associated land use changes that may not have been adequately accounted for in many historical simulations (*95*). Likewise, it has been suggested that in winter, central Eurasia has actually experienced a muted warming since the 1980s relative to model simulations. However, it is unclear if this is a robust discrepancy, as the trends lie within, but on the extreme end of, the range of model results when the uncertainties due to internal variability are taken into account (*96*–*98*). There has been a debate in the literature as to whether this discrepancy in Eurasian cooling could have arisen as a result of models underestimating the Eurasian cooling response to sea ice loss, but whether this is the case remains unclear (*98*–*102*).

Trends in SH wintertime storm track activity, as measured by vertically integrated 2.5- to 6-day band-pass filtered eddy kinetic energy, are a feature where observational uncertainty poses a clear challenge. Chemke *et al*. (*103*) demonstrated a much greater strengthening of the SH storm track in four reanalysis products compared to CMIP6 models, but an updated analysis by (*84*), which uses eight reanalyses as well as a like-with-like comparison in terms of the methods used to derive this metric of SH eddy kinetic energy, demonstrates that the degree of discrepancy depends strongly on the reanalysis dataset considered (their results are reproduced in Fig. 1C). Nevertheless, that study also demonstrated an important role for trends in tropical Pacific SSTs in contributing to the observed strengthening of the SH storm track across the South Pacific, and given the indications that models are not representing tropical Pacific SST trends correctly, it is likely that there is a discrepancy, but its quantification is hampered by observational uncertainty. Trends in the zonal mean jet stream are also an uncertain case. Models are found to adequately capture the poleward shift of the jet streams that has been seen in observations, but there are concerns about whether this is for the right mechanistic reasons. This is because upper tropospheric warming is thought to play an important role in this poleward shift and there are indications that models may not be correctly capturing the relationship between upper tropospheric warming and this poleward shift (*104*). In general, it is expected that in the coming years as the forced signal in circulation increases, there will be much more investigation into whether models are accurately capturing forced regional circulation trends and the mechanisms involved (*105*).

The prior literature has emphasized the nuance and the challenges of confronting ESM trends with the observational record and the multitude of factors that can lead to apparent discrepancies. There are also clear examples where our thinking has evolved over time, such as the example of the warming of the tropical upper troposphere discussed above. There are cases where the models were able to capture trends, but it was then identified to be for the wrong reasons, e.g., the case of observed sea ice trends only being reproduced in models that warm too much (*22*). There are also cases where discontinuities in the forcing datasets have been shown to lead to spurious trends in models (*106*, *107*), and there are cases where more robust procedures for performing a like-with-like comparison with observations have informed our views on a discrepancy (*47*, *108*). There are also, of course, many cases where a misrepresentation of processes in models is probably the dominant issue. The collective assessment of ESM trends is also likely subject to issues of selection bias and multiple hypothesis testing: It is likely easier to publish results that point out a discrepancy as opposed to a success, and the more variables, spatial regions, and trend lengths that are assessed, the more likely it is to find a discrepancy by random chance. These issues speak to the need to fully understand the origins of discrepancies when they are found. To make progress with this problem, it is clear that the research community must ensure that the most robust procedures for assessing model representation of trends are followed, and that we develop tools and methods that can aid in the identification of discrepancies and the understanding of the mechanisms involved, as will be discussed in the following sections.

## ROBUST PROCEDURES FOR CONFRONTING OBSERVED AND MODELED TRENDS

Experience has now taught us that great care must be taken both in identifying a discrepancy between models and observations and in understanding its origins. Below, we outline some proposed best practices, which are based on community feedback, for use when comparing modeled and observed trends.

1) Ensure a “like-for-like” comparison is performed when comparing observations and climate models. Before conducting any comparison, all observational and model datasets should be interpolated to a common temporal and spatial resolution [e.g., horizontal grid and number of vertical levels (*82*)]. Care should be taken when interpolating (e.g., precipitation from stations) to ensure consistency and avoid distortion (e.g., over complex topography or coastlines) and to ensure that an appropriate method and order of operations is used for the field of interest (*109*). For example, for metrics of precipitation variability, it is most appropriate to use a conservative regridding approach and to regrid before calculating the variability metric of interest, although it must be cautioned that even careful regridding alters the statistical properties of precipitation (*109*). To compare models with satellite data, satellite simulators should be used in models such that the model output is directly comparable to observations [e.g., (*110*)]. Similarly, when using observational data that have varying spatiotemporal coverage, model data should be masked to ensure a like-for-like comparison with the observations [e.g., (*47*)]. Identical methods should be used for observations and models, ideally with shared open-source software and documentation so that studies by different authors can calculate quantities consistently.

2) Make use of a large number of observational datasets unless there is robust evidence that they may not be accurate. A large number of available observational datasets should be carefully examined, accounting for user guidance and uncertainty estimates made by data providers and ensuring that any inhomogeneities and nonstationarities in the observed time series are not the result of observing practices, measurement instruments, or spatial/temporal sampling changing over time [e.g., (*47*)]. If possible, multiple physically related quantities should be examined to assess the existence of an observational trend, making sure that physically consistent trends occur in more than one variable [e.g., Deser *et al*. (*111*), who compared SST trends with trends in cloudiness, precipitation, and sea level pressure or, Santer *et al*. (*112*), who related tropical tropospheric warming with other physically related quantities such as tropospheric moistening and sea surface warming]. Ideally, a combination of reanalysis, satellite, and ground-based observations [see, e.g., (*67*)] and/or, where available, observational ensembles that sample observational uncertainty should be used to constrain the magnitude of any observational trends and account for the role of internal variability in those trends. However, if reanalysis is relied on, we recommend focusing on quantities that are tightly constrained by assimilated data [e.g., (*113*)].

3) Compare observations to individual model runs in addition to the ensemble mean. Observed trends are a combination of a forced component and internal variability, and the latter is just one (random) sample of internal variability from many that could have occurred. Thus, observed trends should not be compared directly to the multimodel mean or ensemble mean from one model, which, by definition, average out internal variability and only account for the forced response. Instead, observed trends should be compared to trends from a sufficiently large number of individual model runs, which encompass both the forced response and internal variability (*114*). The use of single-model initial-condition large ensembles (SMILEs) is encouraged [e.g., (*115*, *116*)], and in cases where a large ensemble is used, it is recommended to quantify the magnitude of the discrepancy, including accounting for the uncertainty in observations. Alternatively, methods aiming at isolating the forced response from observations represent promising tools to directly compare forced components (see discussion in the “Cutting-Edge Methods for Identifying Sources of Discrepancies” section), and if SMILEs are not available, sampling of pre-industrial control simulations with an equivalent length to the observational record can give an indication of the uncertainties due to internal variability, providing that the internal variability is not substantially modified by external forcings.

4) Make use of as many different models as possible. When comparing observed trends with those from climate models, simulations from multiple models should be examined to account for model structural differences (unless there is good reason to discount some models). However, when analyzing multiple simulations from multiple models, models with larger numbers of ensemble members could unduly influence the results unless large ensembles are examined individually (as in Fig. 1, A and B), each model is limited to the same number of ensemble members, or weighting is applied to account for differing numbers of ensemble members from each model. Relatedly, it should be ensured that results from multimodel analyses are not unduly influenced by the inclusion of models that are related to one another, as including too many related models (such as those from the same modeling center or sharing particular model components) may not be an accurate representation of the true spread in model structural differences [e.g., (*117*–*119*)]. In some situations, it might be useful to address model independence and the number of ensemble members together using weighting (*120*).

5) Assess whether model simulations have a reasonable representation of internal variability. The analyses discussed above may yield misleading results if the models do not have an accurate representation of the statistical properties of internal variability in the observed climate system. In cases where the observational record is short and the low-frequency climate modes play a role in the uncertainty due to internal variability, this will be challenging to truly quantify but it can at least be checked whether the sampling uncertainty due to high-frequency variability appears correct. The paleoclimate record over the last millennium can also offer some insights into modeled representation of low-frequency modes of both internal variability and naturally forced variations when compared with simulations of the last millennium, if available (*121*). Such datasets do come with challenges of interpretation, particularly for regional features. Observed variability has been used to generate statistical “observational large ensembles,” which can then be used to correct for biases in internal variability in large ensembles from individual models or to validate the spread of large ensembles in models against this statistically derived spread [e.g., (*122*, *123*)]. Ensuring that the width of the distribution of modeled trends that arise as a result of internal variability is a realistic representation of the real world nature of internal variability through comparison to such statistically generated distributions is important to ensure that accurate conclusions are drawn as to whether there is a discrepancy or not. A further challenge, however, is that models may be improperly representing the “signal-to-noise” ratio, as the forced response in modes of internal variability may be unrealistically too weak [e.g., (*124*)], so even in cases where the variability appears correct, it may be for the wrong reasons (*125*).

6) Check for robustness of conclusions to trend length and spatial averaging. It should be ensured that the discrepancies are not heavily influenced by the start and end point of the trend record. The length of observational records does, of course, place limits on the extent to which this can be assessed, but efforts should be made to ensure that similar conclusions are drawn for a range of trend lengths over the record available to ensure robustness of conclusions [e.g., (*126*)]. It should also be ensured that conclusions are not heavily influenced by the choice of spatial average by, for example, showing the full spatial structure of discrepancies in map form.

We conclude this section with some illustrative examples of how these practices have been applied in the recent literature. In the early 21st century, the so-called global warming hiatus occurred, in which warming seemed to pause in observations between 1998 and 2012, while model simulations continued to project increasing temperatures [see e.g., (*127*)]. For this particular problem, applying all available observational temperature datasets (best practice 2) revealed that the hiatus was most severe in the earlier HadCRUT3 dataset. Examining infilling practices for regions of sparse observations—in this case, the Arctic wintertime—turned out to be crucial so that fields were compared in a like-for-like manner with models [best practice 1; (*128*)]. Comparing the observations with individual model ensemble members, rather than the ensemble mean, is the appropriate comparison (best practice 3), which was sometimes overlooked especially in the public discussion. The scientific literature also assessed the representation of internal variability (best practice 5) in the models [e.g., (*129*)], and there was some investigation into the role of differences in forcings between the scenario projections used in models and what actually occurred (*127*, *130*). Idealized climate model experiments in which tropical Pacific SSTs were nudged to observations reproduced the slowdown of warming (*131*). Following these best practices improved our understanding of this temporary slowdown of warming in observations and led to a lower magnitude of discrepancy than previously thought. However, the aforementioned discrepancy in tropical Pacific SSTs is still apparent today and implies that there is more to learn regarding this issue (see Fig. 2).

**Fig. 3.**
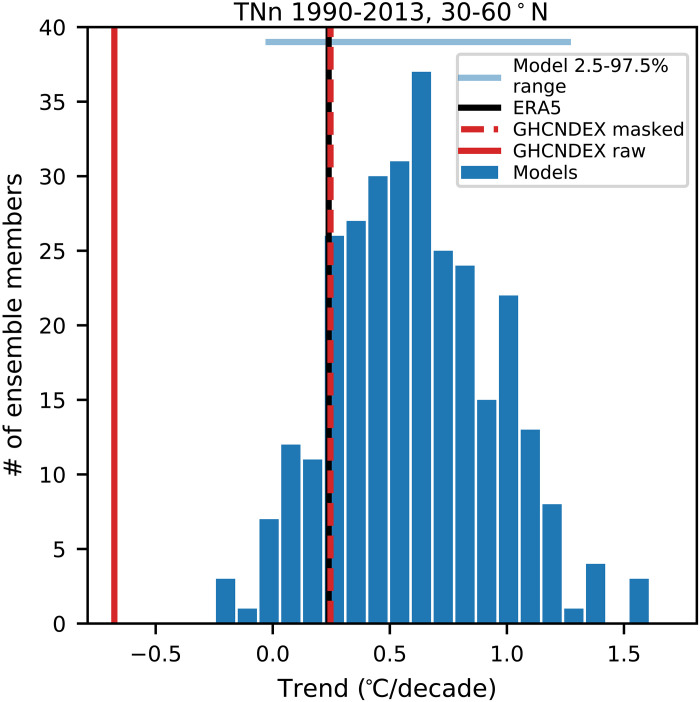
The impacts of changing observational coverage over time on the trends in coldest daily minimum temperatures. Trends in the coldest daily minimum temperature each year (TNn) from 1990 to 2013 and averaged over the mid-latitudes (30°N to 60°N, land only) in observations (red lines), reanalysis (black lines), and 300 historical simulations from seven large ensembles from the CMIP6 era models (blue bars, see Materials and Methods). The solid and dashed red lines show the raw and corrected GHCNDEX datasets, respectively, where the correction applied is to account for changing spatial coverage over time. Adapted from (*47*).

Another example is shown in Fig. 3, taken from (*47*). An earlier study (*48*) reported that the coldest daily minimum temperature in each year was getting colder across the NH mid-latitudes, contrary to expectations in a warming climate and contrary to the range of trends found in models (compare red solid line with the blue histogram in Fig. 3). The use of large ensembles (best practice 3) showed that despite the strong forced warming trend, it was possible (albeit rare) that internal variability could lead to a cooling trend. The use of multiple observational datasets (best practice 2), however, revealed large observational uncertainty (ERA5 compared to GHCNDEX raw in Fig. 3), and efforts to understand this have recently uncovered artifacts in the observed dataset related to changing spatial coverage with a decrease in station coverage over the lower (warmer) part of the mid-latitudes, leading to a spurious trend in the station-based observations. After accounting for these inhomogeneities, the station-based observations align with reanalyses, showing a weak warming trend (GHCNDEX masked in Fig. 3), well within the model ensemble range (*47*).

Most of these best practices have also been used in the context of the discrepancy in tropical Pacific SST trends (see the “Current discrepancies and uncertainties” section). The modeled trends have been compared with many observational SST datasets, and now with an ensemble of observation-based estimates in Fig. 1 (best practice 2). In studies on this topic, the observational record has been compared with individual model simulations (best practice 3), many simulations or large ensembles have been used (*53*, *74*) (best practice 4), and it has been shown that the discrepancy is robust to varying trend start and end points (*126*) (best practice 6).

The goal of applying the best practices is to improve the confrontation process and hopefully bring us closer to an accurate assessment. In doing so, conclusions may go back and forth, as in the case of the magnitude of tropical upper tropospheric warming discussed in the “Successes and partial successes in large-scale thermodynamic trends” section, but the aim is for convergence on a robust and correct answer in the end.

## MOVING BEYOND QUANTIFICATION INTO UNDERSTANDING

Once a discrepancy in trends between models and observations has been firmly established (and observational bias, problems with forcings, and internal variability have been ruled out as explanations), an important next step on the path toward improving ESM predictions is to understand what underlies the discrepancy. Progress in understanding trends in observations and ESMs can be made by utilizing diagnostic frameworks, model hierarchies, and hypothesis testing with climate model experiments. It is important to establish what physical processes are responsible for the trends in observations and ESMs not only when there is a discrepancy between the observed and model predicted trend over the historical period but also in cases where the trend was successfully predicted. It is important to get the right answer for the right reason.

Much has been learned about the physical processes controlling the forced response to anthropogenic climate change. A wealth of literature exists on the mechanistic explanations of the forced response of extremes (*132*, *133*), atmospheric circulation (*134*, *135*), polar climate (*136*), and clouds (*137*). Many of these mechanistic explanations came about by applying diagnostic frameworks, such as radiative feedback and forcing, moist static energy and momentum conservation, and thermodynamic and dynamic decompositions, to simulations of equilibrium and transient responses to anthropogenic climate change. Such frameworks have generally not been applied to historical trends due to the small signal-to-noise ratio, because reanalyses do not necessarily close budgets in a self-consistent way, and because the availability of model data for comprehensive budget analysis is often limited. However, large ensembles help to address the signal-to-noise problem and make it possible to study the dominant physical balances for historical trends in ESMs, and this can also help to contextualize similar mechanistic analyses in reanalysis [e.g., (*138*, *139*)]. For mechanistic analysis of trends in reanalysis, if the budget residual is large, the lack of budget closure needs to be considered as a source of observational uncertainty, following the procedure in the previous section. Cases where the dominant balances differ between observations and models represent opportunities for continuing on the path toward understanding the discrepancy.

The results of diagnostic mechanistic analysis of observed and modeled trends can be used to formulate physical hypotheses to explain trend discrepancies. These hypotheses can then be tested with existing modeling output or dedicated additional model experiments. Such research can help to identify which model improvements and additional observations are most needed.

**Fig. 4.**
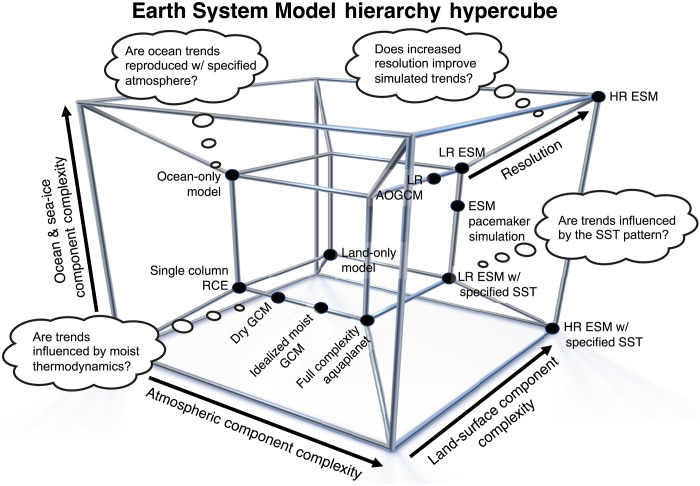
Schematic depiction of the multidimensional ESM hierarchy on a four-dimensional hypercube. It also shows examples of how this hierarchy can be used to understand trends. Each component (e.g., atmosphere, ocean and sea ice, and land surface) has its own hierarchy, and horizontal and vertical resolution can be thought of as additional dimensions in the ESM hierarchy. RCE, radiative-convective equilibrium; GCM, general circulation model; AOGCM, atmosphere-ocean GCM; LR, low resolution; HR, high resolution.

Particularly important for hypothesis testing in the complex and highly coupled climate system is the concept of a hierarchy of models (*140*–*142*). Figure 4 depicts the axes of ESM complexity for the atmosphere, land, and ocean and sea ice components along the sides of the inner cube. Complexity related to enhancements in resolution is depicted by the extension from the inner to the outer cube. A hierarchy of models for the atmosphere could include, for example, a single column model in radiative convective equilibrium, a dry dynamical core, an aquaplanet with idealized or full complexity moist physics, and an atmospheric general circulation model with a land surface model and prescribed SSTs. This hierarchy is depicted on the nearest two sides of the inner cube in Fig. 4, with the left side representing increasing atmospheric complexity and the right side also representing the increase in land complexity between aquaplanet and the atmosphere-land configuration. For the case of confronting ESM trends with observations in particular, it can be useful to switch individual Earth system components, such as the ocean, atmosphere, or land surface, between noninteractive and fully coupled mode, to answer specific questions about the influences on a trend of interest. Such idealized approaches can be useful to gain mechanistic understanding, but care should also be taken to ensure that results are not heavily affected by the unphysical nature of the imposed constraint [e.g., (*143*)]. An example of this approach is the use of simulations that prescribe SSTs from observations either directly as a boundary condition [Atmosphere Model Intercomparison Project (AMIP) or Low Resolution (LR) ESM with prescribed SSTs in Fig. 4] or through nudging SST anomalies in coupled simulations (ESM pacemaker simulations in Fig. 4) to inform on whether trend discrepancies in radiation, circulation, and extreme weather are related to discrepancies in the SST warming pattern (*26*, *78*, *82*, *84*, *131*, *144*). This work has shown that the trend discrepancies in low clouds, radiation, and SH storminess, among others, are likely connected to the well-established discrepancy in the pattern of tropical SST trends (*53*, *74*, *75*, *84*). In this way, experiments with prescribed SSTs can be used to assess whether models do represent the processes of relevance once given the correct historical evolution of SSTs, offering the potential to narrow down the options for potential causes of discrepancies. Similar approaches to constrain models exist for the atmosphere, such as constraining the atmospheric circulation through nudging, or constraining the radiative impacts of clouds through cloud locking techniques (*145*, *146*).

Because of the importance of tropical SST for inducing teleconnections to the extratropics, there has been particular focus on understanding the origins of the tropical SST pattern trend discrepancy (see the “Current discrepancies and uncertainties” section) and its impact on the extratropical circulation trends in the NH and SH [see, e.g., review (*147*)]. One hypothesis for the origins of the SST trend pattern discrepancy is that it is related to mean-state biases in SST and surface fluxes, and this hypothesis has been tested using surface flux–corrected simulations across the model hierarchy (*74*, *148*). Another hypothesis is that the SST trend pattern discrepancy occurs due to trend biases in the Southern Ocean and a too-weak two-way teleconnection between the tropical Pacific and the Southern Ocean, and these hypotheses have been tested using coupled model pacemaker simulations (*82*, *149*, *150*). In both cases, these climate model experiments offer some support for their respective hypotheses, but there remains no definitive answer on the origin of the SST trend pattern discrepancy; it is likely that a combination of mechanisms is needed to fully explain it. An increase in atmospheric and oceanic resolution in the CESM1 model has been shown to alleviate mean-state biases (*151*) and reduce the discrepancy in historical SST trends (*83*), although the reasons for this improvement have not yet been fully understood and could arise from improvements in multiple processes simultaneously as resolution is increased. Applying diagnostic mechanistic analyses to these and other new high-resolution simulations offers a potential boon to the understanding of the SST trend pattern discrepancy.

Comparing trends in the broader Earth system between models and observations requires an expansion of the model hierarchy beyond the traditional atmosphere-ocean model hierarchy of dry dynamical core, moist slab-ocean aquaplanet, atmospheric model with specified SSTs, coupled model pacemaker, and fully coupled model. A schematic depiction of this multidimensional model hierarchy, including different levels of complexity in each Earth system component, is depicted in Fig. 4, with model resolution depicted as a fourth axis of the hypercube. As one example, for studies investigating trends in stratospheric and upper-tropospheric circulation, the hierarchy could be expanded to compare models with high versus low model tops and coupled versus prescribed ozone chemistry [e.g., (*152*)]. For studies investigating trends in the land carbon sink or properties of land surface ecosystems, the hierarchy could be expanded to include perturbed parameter ensembles of the land surface component (*153*) or to explore different complexities of land surface models. Perturbed parameter ensembles are a relatively new tool that are also useful for the atmospheric component given the wide variety of parameter choices that govern the behavior of processes such as aerosol-cloud interaction (*154*, *155*). Finally, model hierarchies can be used to demonstrate a minimal set of processes needed to reproduce a trend, for example, by showing that coupled model trends can be reproduced in aquaplanet simulations [e.g., (*156*)]. These are only a few of many possible uses of model hierarchies for understanding trends and their differences between ESMs and observations; additional hierarchies are available for the ocean, sea ice, and atmospheric parameterization components, as depicted in Fig. 4.

Ultimately, better understanding of trend discrepancies and successful trend predictions relies on a wealth of observations, in addition to novel simulations. It is important to develop a feedback loop where new and existing observations are used to quantify the fidelity of climate model predictions in real time, and where mechanistic understanding from model simulations and analysis can inform and motivate model improvements and the development and maintenance of observational datasets.

## CUTTING-EDGE METHODS FOR IDENTIFYING SOURCES OF DISCREPANCIES

The last decade has seen improvements in observational products and reanalyses, and more modeling centers running large ensembles and single-forcing large ensembles, all of which have improved our capability to evaluate and understand ESM trends through comparison with observations. As we look to the future, we discuss several cutting-edge methods where the potential to understand the sources of trend discrepancies between ESMs and observations has not yet been fully realized: Initialized hindcast systems offer the potential to examine how model errors evolve in a more constrained environment; increasing resolution offers the potential to improve the representation of small-scale processes in the atmosphere and ocean; and artificial intelligence and machine learning (AI/ML) is an emerging area that could aid in a number of areas of relevance to evaluating model trends.

### Initialized hindcasts

Climate models are often the same, or very similar, to models used for initialized near-term prediction, for example, on seasonal or decadal timescales. This opens up an important avenue along which to explore model uncertainties, in particular through analysis of hindcast datasets. These are routinely produced for the purposes of forecast validation and consist of a series of short simulations attempting to “forecast” past years or seasons from observation-based initial conditions. Such hindcast sets offer several advantages for studying model behavior as they (i) typically cover the recent few decades, (ii) are often relatively high-resolution coupled modeling systems, (iii) have smaller initial biases due to the frequent re-initialization, (iv) can be validated against observations in a forecasting sense, and (v) are also constrained by the initialization to track the observed system in terms of large-scale modes of variability (e.g., El Nino Southern Oscillation, Atlantic Multi Decadal Variability, and Pacific Decadal Oscillation). They can be used for studying trends by simply considering the forecasts as a set of simulations of individual seasons or years, and then taking the multidecadal trend over this set, although challenges could arise if the drift of these models from the observation-based initial state is not stationary in time (*157*). Despite these challenges, we argue that this presents a promising resource for investigating discrepancies in recent trends.

Several important climate model biases have been found to develop on relatively short timescales in forecast mode, sometimes within hours [e.g., Southern Ocean cloud biases; (*158*)]. Likewise, prediction systems have been found to feature errors in multidecadal trends, which develop during the simulation period of each short, initialized forecast (*159*). Beverley *et al*. (*160*) demonstrated striking similarity between ensemble mean trend errors in seasonal prediction systems and CMIP6 models in a range of variables including SST, precipitation, and atmospheric circulation. They interpreted the rapid development of these errors as a signal of the importance of atmospheric processes, and further suggested that this indicates that the model’s mean-state bias is not independent of the external forcing.

Another application of seasonal hindcasts exploits the spread in these ensembles over the hindcast period to provide estimates of plausible recent trends that arise from both the predictable signal and the unpredictable noise, analogous to the way in which hindcast simulations have been used as plausible event sets to inform on present-day climate risks of extreme events (*161*). Thomas *et al*. (*162*) used a long hindcast set to study simulated trends since 1980, finding striking differences in ensemble spread in the system. Amplified Arctic warming trends, for example, are surprisingly unconstrained by initialization, although patterns such as AMV and PDO follow the observations closely. In contrast, while there is some spread in mid-latitude jet trends, the hindcasts add confidence to the emergence of a general poleward jet shift on the global scale (*104*).

### Higher resolution

Climate model resolution has gradually increased over the past several decades, from a typical horizontal resolution of roughly 500 km at the time of the IPCC 1st Assessment Report to roughly 200 km at the time of the IPCC 3rd Assessment Report and roughly 100 km at the time of the IPCC 5th Assessment Report. Many modeling centers are now exploring the benefits of even higher horizontal resolution of O(10 km) or O(1 km). This poses opportunities for identifying sources of discrepancies in climate model trends, because these high-resolution models reduce the reliance on parameterizations, which are the primary source of model structural uncertainty (*163*).

**Fig. 5.**
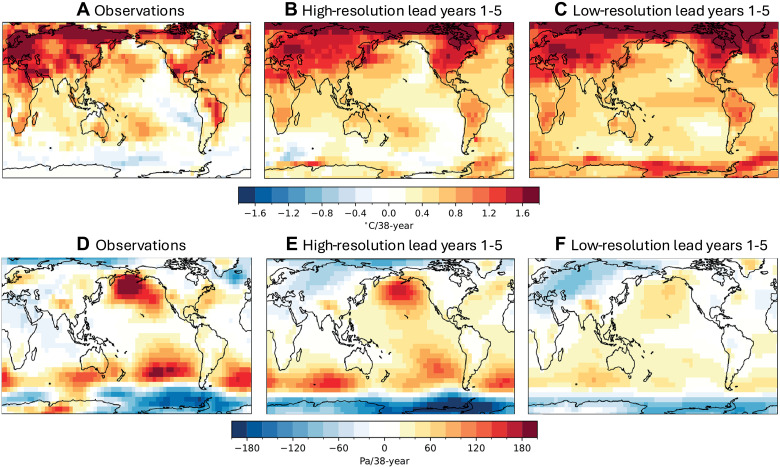
Comparing trends in high-resolution and low-resolution initialized predictions. Reproduced from (*83*), their figure 6, this shows global trends in annual averaged near-surface air temperature for 1979 to 2017. (**A**) Trends in near-surface air temperature/SST trends in observations [using CRU-TS (*212*) over land, and HadISST (*206*) over ocean]. (**B**) Trends for the ensemble mean of 10 members of initialized predictions with high-resolution (~0.25° atmosphere, ~0.1° ocean) simulations with CESM1. (**C**) Same as in (B) but for low-resolution (~1° atmosphere and ocean) CESM1. The CESM1 trends are computed from the average of lead years 1 to 5 after a November 1st initialization from observation-based ocean and sea ice states. For comparison to the lead year 1 to 5 average trends, a 5-year running smoother is applied to the observed time series before the computation of the trend. (**D**) to (**F**) are as (A) to (C) but for sea level pressure, using ERA5 for observations.

The highest resolution models for which full CMIP-type historical simulations have been run have roughly 25-km horizontal resolution in the atmosphere and roughly 10-km horizontal resolution in the ocean (*164*), allowing better representation of ocean mesoscale eddies, atmospheric fronts, and topography than in typical O(100 km) resolution CMIP6 models. Of these, only one that we are aware of [CESM1-HR, (*151*)] has released data from multiple ensemble members over the historical period, as is needed to distinguish between forced responses and internal variability. This model shows historical trends in SST, atmospheric circulation, and precipitation that look more like observed trends and improved multiyear prediction skill for initialized hindcasts (*83*). One of the results from the CESM1-HR initialized hindcasts taken from (*83*) is shown in Fig. 5, and this illustrates both the potential impacts of resolution and provides an example of the use of initialized hindcasts in exploring the model sensitivity of long-term trends. The observed trends in surface air temperature from 1979 to 2017 (Fig. 5A) exhibit the cooling features in the Southern Ocean and Eastern tropical and southern subtropical Pacific that have been discussed above as being distinct from trends that free running coupled simulations produce. Figure 5 (B and C) shows the trends from 1979 to 2017 for the lead year 1 to 5 averages of the ensemble mean of 10 member ensembles of initialized hindcasts with CESM1 at high and low resolution, respectively. The higher-resolution hindcasts exhibit less warming of the Southern Ocean and the Eastern Tropical Pacific, bringing the modeled trend closer to observed. The high-resolution hindcasts also produce sea level pressure trends that are much closer to observed (Fig. 5, D to F). Both initialization and model differences could play a role in the differences between these trends, but they offer a preliminary indication that increasing resolution could make a difference and also suggest that initialized prediction may offer a test bed for understanding processes that are of relevance to long-term forced trends.

It remains to be seen whether the same conclusions hold for other models at this resolution, and as additional simulations at this resolution emerge in the coming years, a lot can be learned from systematically comparing trends between these high-resolution models, their low-resolution counterparts, and observations, and using the diagnostic and model hierarchy approaches outlined in the previous section to understand the physical mechanisms behind any trend differences. Many modeling centers are now developing and running even higher-resolution models, with atmospheric resolution of 1 to 10 km (the so-called km-scale) such that the parameterization of deep convection can be turned off (*7*, *165*–*168*). We note, however, that while computational capability is ever on the rise, it is nonetheless finite. This means that there is a practical trade-off between increasing resolution and simulation length or the number of members in an ensemble, and that experimentation with high resolution will be available to only a small subset of users with access to large amounts of computing time. One possible intermediate solution is variable resolution models [e.g., (*169*, *170*)], which can be used to investigate where increased resolution can alter the behavior of the large-scale system. Regular evaluations of the costs and benefits of increasing resolution [e.g., (*115*)] are important to ensure the best use of community resources.

However, targeted studies of how and why mean-state biases are modified at the km-scale can give insight into the origins of trend biases that are related to biases in the mean state. Furthermore, studying the development of biases in short-term hindcasts with km-scale models could give insight into whether some of the biases that develop in short-term hindcasts with coarser-resolution simulations could be alleviated with higher resolution. If there are improvements in mean-state biases or short-term hindcasts, then machine learning parameterizations trained on km-scale simulations represent a promising avenue for incorporating these improvements in coarser-resolution simulations.

### Artificial intelligence and machine learning

AI/ML are pushing the frontiers of climate modeling (*171*), and they can also contribute to identifying sources of trend discrepancies. As highlighted so far in this review, a key challenge in confronting ESM trends with observations is diagnosing the forced and unforced contributions to the particular realization of the real world that has been observed. Methods of varying complexity, ranging from classical regression approaches to neural networks, have been used to identify the climate change signal from among the noise of internal variability in observations. Examples include dynamical adjustment (*172*, *173*), linear inverse models (*174*), low-frequency component analysis (*175*), AI/ML, or regression models (*176*–*179*). These methods have been shown to reduce the number of ensemble members needed to estimate the forced response and to allow an estimate of the forced response from single realizations (*173*, *175*, *179*). This allows comparison of ESM trends with observations even when large ensembles are not available and reduces the contribution of internal variability to any remaining trend discrepancy. The Forced Component Estimation Statistical Method Intercomparison Project (ForceSMIP) is currently comparing 30 different methods for isolating the forced response from single realizations; they find that novel AI/ML methods perform comparably or slightly better than existing statistical methods, but it is worth noting that these AI/ML methods have potential for further improvement. A related potential application of AI/ML methods is in identifying contributions from different external forcings (e.g., greenhouse gasses, anthropogenic aerosols, and volcanoes) to historical climate change, which could build on existing methods for detection and attribution [e.g., (*180*, *181*)].

In addition to the usage of AI/ML in climate data analysis, the utility of AI/ML for improving climate models is also being explored, with different applications using it either to parameterize unresolved processes (*182*–*184*) or to completely replace entire components of the ESM (*185*, *186*). AI/ML are also actively being used to help improve the estimation of unconstrained model parameters within existing parameterizations (*187*, *188*) or to learn model errors to develop bias correction schemes (*189*). Last, AI/ML has the potential to aid in the generation of larger ensemble sizes through emulation of model or observation-based data (*190*), which could aid in the statistical comparison between models and observations, and to also help improve the observational record used for comparison to models by providing approaches to infill or interpolate sparse observational datasets for comparison with ESMs [e.g., (*191*)].

## FUTURE CONFRONTATIONS

We conclude with a discussion of potential priorities and future opportunities for the climate science community over the next few decades. We anticipate that the upcoming decades will be a critical time for climate prediction as anthropogenic signals emerge in more parts of the climate system and the fidelity of climate predictions is quantified in real time. However, for some features, such as coupled ocean-atmosphere changes, it may still remain difficult to separate anthropogenic climate change from internal decadal-to-multidecadal variability in the climate system.

One critical priority over the upcoming decades will be maintaining and improving long-term climate observational records. This includes paleoclimate reconstructions, which can provide an additional constraint on ESM behavior, e.g., (*192*). Long-term continuous observational records are essential for monitoring the emergence of climate change, quantifying decadal variability, and evaluating models. Possible future gaps in critical observational records, such as the Earth’s TOA radiation budget, would be a serious setback for the climate science community [e.g., (*193*, *194*)]. We recommend that climate modelers regularly advocate for maintenance of the existing observational records that are most critical for climate model development and work closely with observational experts to develop new datasets that would be particularly useful for climate model evaluation. Additionally, we recommend that all climate observational records clearly document uncertainty estimates with language that is accessible to nonspecialists so that any comparison of climate model trends with observations is informed by the appropriate observational uncertainty estimates. Finally, while the next decade of data will be especially useful for quantifying trends, new techniques should be explored to take advantage of more existing historical observations to reconstruct additional climate records back in time to provide longer-term context [e.g., (*195*)].

Another key opportunity for the climate science community will be to improve the existing model-observational comparison cycle. At present, the CMIP coordinates a large set of climate model experiments conducted by global modeling centers approximately every 7 years, which are subsequently analyzed by climate scientists worldwide. The CMIP process has resulted in a wealth of knowledge on the climate system, but it is a substantial burden on global modeling centers and many of the discrepancies between observed and historical trends have persisted across the CMIP generations. In this era of rapidly evolving climate, a more frequent, smaller set of experiments, alongside a larger interval between large CMIP efforts, which would allow more time for substantial model development, may be of greater value to the climate science community. This smaller, more frequent, set of experiments could include extending historical radiative forcing and prescribed SST simulation on a yearly basis, as proposed by the CERESMIP project (*196*). These frequently updated experiments would allow for a near real-time assessment of trends and more timely identification of potential climate model biases, which are crucial to detect and address quickly in a period of a rapidly changing climate. More effort should also be made to account for uncertainty in historical radiative forcing, such as running experiments with multiple plausible historical forcings from 1850 to present-day, given that incorrect historical forcings, such as from aerosols, input into models can lead to apparent discrepancies between model and observed trends [e.g., (*72*, *106*, *107*)]. Finally, and perhaps most importantly, improved communication is needed between climate analysts and model developers to ensure that model development choices are made with a view toward understanding, and ultimately reducing, known discrepancies in trends between models and the observational record. A particular challenge is how to bring in knowledge from many multimodel evaluation studies to influence model development at individual modeling centers.

Using a broader array of methods as discussed in the “Robust Procedures for Confronting Observed and Modeled Trends” and “Moving Beyond Quantification into Understanding” sections will also facilitate a more thorough comparison of model and observed trends, allowing for improved understanding of the mechanisms underlying the differences. Emerging computational technologies will allow for higher-resolution models, increasing data storage volumes to store diagnostics for more complex processes in models, machine learning–aided model development, and an increased use of perturbed parameter experiments or initialized hindcasts. At the same time, even with these emerging technologies, the climate science community should continue to rely on a hierarchy of theory and modeling to understand discrepancies between observed and model trends, as differences between observations and models are often best understood within the context of a simplified model (e.g., large-eddy simulations, quasi-geostrophic models for mid-latitude dynamics, simplified ocean processes including sea ice, and simplified land surface models).

As more Earth system processes become incorporated into models, new opportunities will present themselves for confronting trends in these processes with observations. Processes such as evolving ecosystem demography, the global carbon cycle, ice sheets, and glaciers have large societal importance, but have traditionally been absent or poorly represented in models. Some of these processes are currently not well observed so, as modeling of these processes continues to improve, there will be a greater need for observational records of these fields to evaluate the fidelity of models in representing trends in these Earth System processes. However, existing observational datasets of these quantities are often sparse (e.g., ground-based flux towers), and trends can be highly uncertain and dataset-dependent [e.g., soil moisture; (*197*)]. For other quantities, such as land carbon fluxes, there remains no true observational-based estimates. Improving observational constraints of the carbon cycle is particularly important, as future CMIP generations will rely more on emission-driven simulations (*198*), meaning that a realistic representation of the global carbon cycle will be necessary to produce an accurate change in carbon dioxide concentrations and the associated radiative forcing. As models rapidly expand in complexity, it will remain difficult to confront emerging Earth system trends in models with observations without accompanying advancements in long-term Earth system observations.

Overall, as we anticipate increasing emergence of forced trends in the climate system over the coming decades, it seems imperative to be able to track trends and identify discrepancies between observations and models in near real-time. One possible way to do this would be to issue an annual “state-of-the-signal” summary assessment on the current knowledge of historical trends and the ability of climate models to reproduce them. This could build on the current “State of the Climate” published annually in the *Bulletin of the American Meteorological Society*, but would focus more on the comparison of modeled and observed trends. Another avenue would be through an internet-based forum like existing real-time observational monitoring (https://climatereanalyzer.org/). Any such resource would be most beneficial if it could be updated in near real-time and accompanied by appropriate documentation to aid in its interpretation by nonspecialists, including explaining how anthropogenic forcing, internal variability, and observational biases can all contribute to trends in observational datasets. To be most useful, near real-time trend metrics would need to be statistically robust and not overly sensitive to endpoints, and new methods are being developed in this area (*199*, *200*). Reliable real-time information would help the research community to prioritize, for example, balancing the need for long-term work on stubborn problems with the need to understand new, often extreme events as they arise.

In summary, now is a critical time for the climate science community to expand our assessment and understanding of the representation of forced climate signals in ESMs to ensure accurate future projections of the climate system. Lengthening observational records, growing forced signals, and enhanced complexity of models along with technological advances result in the field being primed to rise to this challenge.

## MATERIALS AND METHODS

### Methods for figures

The following list summarizes the models and members (in [ ]) used for the CMIP6 histograms in Figs. 1 and 2, with the first, second, and third numbers depicting the number of members used for panels (A), (B), and (C), respectively:

ACCESS-CM2[3,3,1],ACCESS-ESM1-5[40,40,1],AWI-CM-1-1-MR[1,1,-],BCC-CSM2-MR[1,1,1],CAMS-CSM1-0[-,-,1],CESM2[-,-,1],CESM2-FV2[-,-,1],CESM2-WACCM[3,3,1],CESM2-WACCM-FV2[-,-,1],CIESM[1,1,-],CMCC-CM2-HR4[-,-,1],CMCC-CM2-SR5[1,1,1],CMCC-ESM2[1,1,1],CNRM-CM6-1[5,5,-],CNRM-CM6-1-HR[1,1,-],CNRM-ESM2-1[5,5,-],CanESM5[25,25,1],E3SM-1-1[-,1,-],E3SM-1-1-ECA[-,1,-],EC-Earth3[58,24,1],EC-Earth3-AerChem[-,-,1],EC-Earth3-CC[-,-,1],EC-Earth3-Veg[8,8,1],EC-Earth3-Veg-LR[3,3,1],FGOALS-f3-L[1,1,1],FGOALS-g3[4,4,1],FIO-ESM-2-0[3,3,-],GFDL-CM4[1,1,1],GFDL-ESM4[1,1,-],GISS-E2-1-G[5,5,-],GISS-E2-1-H[5,5,-],HadGEM3-GC31-LL[4,4,-],HadGEM3-GC31-MM[4,4,-],IITM-ESM[1,1,1],INM-CM4-8[1,1,1],INM-CM5-0[1,1,1],IPSL-CM6A-LR[7,7,1],KACE-1-0-G[3,3,1],KIOST-ESM[1,1,-],MIROC-ES2L[10,10,-],MIROC6[50,50,1],MPI-ESM-1-2-HAM[-,-,1],MPI-ESM1-2-HR[2,2,1],MPI-ESM1-2-LR[30,30,1],MRI-ESM2-0[5,5,1],NESM3[-,-,1],NorESM2-LM[1,1,1],NorESM2-MM[1,1,1],SAM0-UNICON[-,-,1],TaiESM1[1,1,1],UKESM1-0-LL[5,5,-]

A dash indicates that a model was not used in a given panel. In addition to these CMIP6 models, the dots in panels (A) and (B) show the following large ensembles from bottom to top: the CMIP5 era large ensembles CanESM2[50], CESM1-CAM5[40], CSIRO-Mk3-6-0[30], GFDL-CM3[20], and MPI-ESM[100] and the CMIP6 era large ensembles ACCESS-ESM1-5[40], CanESM5[25], EC-Earth3[58 for panel (A), 24 for panel (B)], MIROC6[50], MPI-ESM1-2-LR[30], and CESM2[100]. For the CMIP5 simulations, the historical simulations before 2006 are combined with the RCP8.5 scenario thereafter, and for the CMIP6 simulations, the historical simulations before 2014 are combined with the SSP5-8.5 ensembles thereafter. The only exceptions to this were that the SSP2-4.5 scenario was used for the EC-Earth3 ensemble in panel (B) and the CESM2 large ensemble was run under the SSP3-7.0 scenario. For eddy kinetic energy (EKE) in panel (C), which requires high-frequency data, only the first 50 members of the CESM2 large ensemble are shown.

For observation-based data in Fig. 1A, we use ERA5 (*201*), MERRA2 (*202*), JRA3Q (*203*), and NCEP2 (*204*) reanalyses. In Fig. 1B, we use SSTs from ERSSTv5 (*205*), HadISST (*206*), and the 200-member DCENT ensemble (*180*). For Fig. 1C, all data were taken from (*84*) and readers are referred to that study for the methods.

For Fig. 2, the models and members are the same as those used in Fig. 1C. SST (ts) was used over ocean grid points, and surface air temperature (tas) was used over land grid points for CMIP6. The models were first interpolated to a 1° grid before assigning grid points as land or ocean using a 1° resolution land mask. The observational datasets used in Fig. 2 are the 200-member DCENT ensemble along with the BEST (*207*), GISTEMP (*208*, *209*), and HadCRUT (*210*) surface temperature datasets, which represent surface temperature over the ocean and surface air temperature over the land.

Figure 3 was adapted from (*47*) and uses 300 historical simulations from seven large ensembles from the CMIP6 era: ACCESS-ESM1-5[40], CanESM5[50], CESM2[50], EC-Earth3[50], GFDL-SPEAR-MED[30], MIROC6[50], and MPI-ESM1-2-LR[30]. The masked GHCNDEX (*211*) data only include grid points that have complete temporal coverage (*47*).
